# High yield of culture-based diagnosis in a TB-endemic setting

**DOI:** 10.1186/1471-2334-12-218

**Published:** 2012-09-14

**Authors:** Anne-Marie Demers, Suzanne Verver, Andrew Boulle, Robin Warren, Paul van Helden, Marcel A Behr, David Coetzee

**Affiliations:** 1 Département de Microbiologie et Immunologie, Université de Montréal, Montreal, QC, Canada; 2 CHU Sainte-Justine, 3175 Chemin de la Côte Sainte-Catherine, Montréal, QC, H3T 1C5, Canada; 3 KNCV Tuberculosis Foundation, The Hague, The Netherlands; 4 CINIMA, Academic Medical Centre Amsterdam, Amsterdam, The Netherlands; 5 Centre for Infectious Disease Epidemiology and Research, School of Public Health and Family Medicine, University of Cape Town, Cape Town, South Africa; 6 DST/NRF Centre of Excellence for Biomedical TB Research/ US/MRC Centre for Molecular and Cellular Biology, Division of Molecular Biology and Human Genetics, Department of Biomedical Sciences, Faculty of Health Sciences - Stellenbosch University, PO Box 19063, Tygerberg, 7505, South Africa; 7 Department of Medicine, McGill University, Montreal, Québec, Canada; 8 McGill University Health Centre, Room A5.156, 1650 Cedar Avenue, Montreal, QC, H3G 1A4, Canada

**Keywords:** Tuberculosis, Diagnosis, Culture, Microscopy

## Abstract

**Background:**

In most of the world, microbiologic diagnosis of tuberculosis (TB) is limited to microscopy. Recent guidelines recommend culture-based diagnosis where feasible.

**Methods:**

In order to evaluate the relative and absolute incremental diagnostic yield of culture-based diagnosis in a high-incidence community in Cape Town, South Africa, subjects evaluated for suspected TB had their samples processed for microscopy and culture over a 21 month period.

**Results:**

For 2537 suspect episodes with 2 smears and 2 cultures done, 20.0% (508) had at least one positive smear and 29.9% (760) had at least one positive culture. One culture yielded 1.8 times more cases as 1 smear (relative yield), or an increase of 12.0% (absolute yield). Based on the latter value, the number of cultures needed to diagnose (NND) one extra case of TB was 8, compared to 19 if second specimens were submitted for microscopy.

**Conclusion:**

In a high-burden setting, the introduction of culture can markedly increase TB diagnosis over microscopy. The concept of number needed to diagnose can help in comparing incremental yield of diagnosis methods. Although new promising diagnostic molecular methods are being implemented, TB culture is still the gold standard.

## Background

For years, the World Health Organization (WHO) and the International Union against Tuberculosis and Lung Disease (IUATLD) recommended sputum smear microscopy as the cornerstone to diagnose pulmonary TB, as smear-positive subjects are most contagious [1,2]. However, since smear microscopy has a detection limit of about 5000–10 000 organisms/mL [3] - compared to 10–100 organisms/mL for culture [4] - specimens with < 5000 bacteria are negative by microscopy.

In settings where microscopy and culture are both done, such as developed countries, approximately half of culture positive specimens have negative smears [5-8]. Based on this, one anticipates a doubling of case detection if culture methods are introduced into a setting that only does microscopy.

WHO recommends the use of liquid culture in low income settings where feasible [9]. However, other authorities consider the role of culture to be primarily for surveillance and diagnosis of MDR-TB [10]. Little is known on the yield of culture in the context of national TB control programmes in high incidence countries. We have evaluated the relative (RY) and absolute yield (AY) of introducing culture-based diagnosis in a high incidence setting (Cape Town, South Africa).

## Methods

### Study design

#### Prospective cohort study in regular programme setting

##### Study setting

This study was conducted in Gugulethu, a residential area in Cape Town, South Africa, as part of a molecular epidemiology study. At the time of the study, the notified incidence of TB was 992/100 000 and the antenatal HIV prevalence was 28.1% [11]. The setting is still similar now with respect to incidence rate and proportion of patients HIV infected [12].

#### Inclusion criteria

From 1 April 2002 to 31 December 2003, subjects older than 14 years presenting to the NY1 Gugulethu TB clinic with signs or symptoms of pulmonary TB were considered suspects and included in the study. Because some individuals presented more than once during the study period, we analyzed the data by suspect episodes (SE) and not patients. We defined a new SE if the same person returned to the clinic more than 6 weeks after a previous evaluation. A previous analysis describing a method to assess laboratory cross-contamination has already been published [13].

#### Data collection

Routine diagnosis and treatment procedures were followed by clinical staff, except that culture was added to the smear-based diagnosis recommended by the South African TB Guidelines, which restrict cultures to retreatment cases [14]. Every effort was made to collect 2 sputum samples for each SE. Additional smears and cultures were done if judged necessary by the clinic staff. Chest x-rays were not done routinely as per national guidelines.

Demographic and laboratory data were collected for all SE. No supplementary procedures were performed on patients. For treatment episodes, outcome information was obtained from the clinical notes, patient interviews and the TB register. Patients started on TB treatment were interviewed by the study team after informed consent was obtained. Since some patients might have been treated at another TB clinic in the area, we manually matched by name and demographic data the untreated positive culture SE with the sub-district electronic TB register for July 2002 to Oct 2005.

HIV voluntary counseling and testing was offered to every patient put on TB treatment. HIV information was only available for those started on treatment and was collected from patient interviews and record reviews. At the time of the study, antiretrovirals were not routinely available for HIV positive patients. The protocol was approved by the research ethics committee of the Faculty of Health Sciences, University of Cape Town (REC REF 048/2001).

#### Laboratory procedures

Sputum specimens were collected at the Gugulethu clinic and kept refrigerated until transported later the same day to the National Health Laboratory Services (NHLS) in Cape Town where they were processed for fluorescence (auramine) microscopy and culture. Smear results were reported as per WHO/IUATLD classification [15]. Cultures were done both on one Löwenstein-Jensen and one BACTEC MGIT 960 ® bottle (BD Diagnostic Systems, Sparks, MD) and incubated for 8 weeks. If either culture method was positive, the sample was reported positive. Unfortunately, the results for solid and liquid media were not recorded individually. In addition to biochemical methods, positive cultures were speciated as *M. tuberculosis* complex using the Accuprobe® (Gen-Probe Inc., San Diego, CA). Susceptibility testing for isoniazid (INH) and rifampicin (RIF) was done using the indirect proportion method. Details on the laboratory procedure have been described elsewhere [13].

#### Definitions

Only diagnosis specimens were analysed. We considered scanty smears to be positive [16-18]. SE with culture results other than negative or positive for *M. tuberculosis* (i.e. non-tuberculous mycobacteria (NTM), lost viability or contaminated) were excluded from the analysis as it was not possible to assume with certainty that they were negative or positive. For each SE, both smear and culture results were ordered by timing of specimen collection and classified according to the following patterns of successive specimens: N (N = negative), P (P = positive), NN, NP, PP, PN, NNN, NNP, NPN, NPP, etc. Smear and culture results were then combined together per SE. A SE was classified as smear positive if any of the smears was positive. A SE was classified as culture positive if any of the cultures was positive for *M. tuberculosis* complex. Smear and culture results were then grouped together, defining SE as SNCP (smear negative culture positive), SPCP (smear positive culture positive), SPCN (smear positive culture negative) or SNCN (smear negative culture negative).

Because our study was done under routine conditions, not all TB suspects had 2 specimens collected. In order to correctly calculate the incremental yields, we added the observed proportions of positive smears or cultures to the expected proportion for the missing specimens, using a method described by Rieder et al. (Appendix)[19]. Since less than 10% of SE had a 3rd smear or culture done, we could not calculate accurately the yields for the third specimen. We therefore restricted the analysis to SE with ≤2 smears & ≤2 cultures. We also regrouped our initial patterns of successive specimens into Rieder’s patterns, adapted for 2 specimens: N9 (where N is negative and 9 is a missing specimen), NN, NP and Px (where P is positive and x is a subsequent result of no interest for the calculation of incremental yields) (Appendix)[19].To calculate the relative and absolute yield of smears and cultures, we used the formulas shown in Table 1. We defined the number needed to diagnose (NND) as the reciprocal of the absolute yield. We did not use another proposed definition for NND [20] because it did not consider prevalence [21].

**Table 1:** Definitions

**Definition**		**Formula**
Relative incremental yield of method* 1 vs method 2 (RY)	=	% positive per method 2
		% positive per method 1
Absolute incremental yield of method 2 vs method 1 (AY)	=	% positive per method 2 - % positive per method 1
Number needed to diagnose one extra case of TB (NND)	=	1
		AY (in %)

**Legend:** *Method: can either be 1 smear, 2 smears, 1 culture, 2 cultures.

#### Data analysis

Data were collected and analyzed using Microsoft Access 2002 database and Stata software, version 10 (Stata Corporation). The chi-squared test was used to compare proportions.

## Results

**Figure 1:**
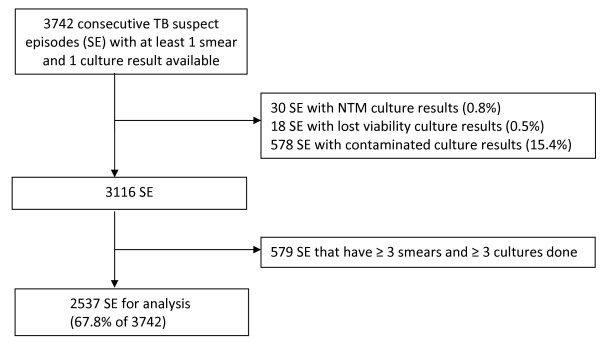
**Study flow diagram.** Legend. SE = Suspect Episodes, NTM = Non-tuberculous mycobacteria.

During the study period, 3742 SE had at least 1 smear and 1 culture done. After excluding the SE which were contaminated, showed the presence of a NTM or lost viability, and after removing the SE with ≥3 smears or cultures, 2537 SE were left for analysis (67.8% of 3742) (Figure 1). These 2537 SE represented 2207 individuals: 62% were male with a median age of 35 years (interquartile range 27–45). Of note, only 1 of the 30 excluded SE with NTM had a mixed infection with *M. tuberculosis*.

### Smears and cultures results

In Table 2, smear and culture results are presented for the first and for both specimens (combined field). The sensitivity of smear compared to culture was 50.6% and 58.9% respectively. Of the 2537 SE, 20.0% had at least one positive smear and 29.9% had at least one positive culture after 2 specimens (expected proportion of positives in Table 3). Using all smear positive SE as a denominator, the fraction of SE positive on the first smear (Fd1) was 74.3% and the incremental yield (IY) of the second smear (Fd2) was 25.7% (Table 3). For cultures, 89.8% of positive cultures were positive on the first specimen, and the IY of the second culture was 10.2%. The number of specimens needed to find one case on the first smear was 7. When comparing the second smear to the first smear, 19 specimens would need to be examined before finding another positive one. For cultures, only 4 specimens would be needed to find a positive result on the first specimen but when comparing the yield of the second culture to the first culture, the NND would be 33. Table 4 shows the results presented as RY, AY and NND to compare different screening strategies. The relative yield of doing 1 culture was about twice the yield of 1 smear (1.8), for an absolute yield of 12.0%, meaning that in this setting, one can detect one additional case of TB by performing 8 additional cultures (NND = 8). In contrast, one additional case was diagnosed when 19 patients submitted a second sample for smear microscopy (Tables 3 and 4).

**Table 2:** **Smear and culture results per SE for the 1 **^**st **^**specimen and for both specimens **

**1st specimen**		**Culture**			**After 2 specimens**		**Culture (combined)**		
		**P**	**N**	**Total**			**P**	**N**	**Total**
Smear	P	345	32	377	Smear (combined)	P	432	45	477
	N	337	1823	2160		N	301	1759	2060
	Total	682	1855	2537		Total	733	1804	2537
	Se =	SPCP / CP	50.6%			Se =	SPCP / CP	58.9%	
	Sp =	SNCN / CN	98.3%			Sp =	SNCN / CN	97.5%	
	PPV =	SPCP / SP	91.5%			PPV =	SPCP / SP	90.6%	
	NPV =	SNCN / SN	84.4%			NPV =	SNCN / SN	85.4%	

**Legend:** S = smear, C = culture, P = positive, N = negative, Se = Sensitivity, Sp = Specificity, PPV = Positive predictive value, NPV = Negative predictive value.

**Table 3:** Smear and culture results for all SE, smear negative and smear positive SE

**Description**	**All smears**	**All cultures**	**Cultures in SN SE**	**Cultures in SP SE**
Number of SE	2537	2537	2060	477
Observed # of P SE after 1 specimen	377	682	253	429
Observed # of P SE after 2 specimens	477	733	301	432
Proportion of P SE on 1^st^ specimen	0.149	0.269	0.123	0.899
Proportion of NP SE	0.060	0.042	0.040	0.103
# of P SE missed by failing to do 2^nd^ specimen	31	27	25	2
Expected # of P SE after 1 specimen	377	682	253	429
Expected # of P SE after 2 specimens	508	760	326	434
Expected proportion of P SE after 1 specimen	0.149	0.269	0.123	0.899
Expected proportion of P SE after 2 specimens	0.200	0.299	0.158	0.910
Potential IY for 1^st^ specimen	0.743	0.898	0.776	0.989
Potential IY for 2^nd^ specimen	0.257	0.102	0.224	0.011
Overall fraction of P on 1^st^ specimen	0.149	0.269	0.123	0.899
Overall fraction of P on 2^nd^ specimen	0.052	0.031	0.035	0.010
NND on 1^st^ specimen	7	4	8	1
NND on 2^nd^ specimen	19	33	28	96

**Legend:** S = smear, P = positive, N = negative, SE = Suspect Episodes, NND = number needed to diagnose (see Methods for details), IY = incremental yield.

**Table 4:** Relative and absolute incremental yields and NND for smear and cultures

		**Relative incremental yield vs.**		
**Yield**		**1st S**	**2 S total**	**1st C**
Expected # of P after 1 S = 377/2537 = 14.9%				
Expected # of P after 2 S = 508/2537 = 20.0%		1.3		
Expected # of P after 1 C = 682/2537 = 26.9%		1.8	1.3	
Expected # of P after 2 C = 760/2537 = 29.9%		2.0	1.5	1.1
		Absolute incremental yield vs.		
Yield		1st S	2 S total	1st C
Expected # of P after 1 S = 377/2537 = 14.9%				
Expected # of P after 2 S = 508/2537 = 20.0%		5.2%		
Expected # of P after 1 C = 682/2537 = 26.9%		12.0%	6.9%	
Expected # of P after 2 C = 760/2537 = 29.9%		15.1%	9.9%	3.1%
		NND vs.		
	NND	1st S	2 S total	1st C
1st Smear	7			
2 Smears total	5	19		
1st Culture	4	8	15	
2 Cultures total	3	7	10	33

**Legend:** S = smear, P = positive, N = negative, SE = Suspect Episodes, NND = number needed to diagnose (see Methods for details), IY = incremental yield. Note: Expected # of P after 1 S or C is same as Observed (see Appendix for details).

We compared the culture results of the smear positive SE to the smear negative SE (Table 3). Smear positive specimens are expected to be culture positive so yields are presented mainly as a comparison to the smear negative SE. Although the potential IY for the first culture was lower at 77.6% compared to all cultures (89.8%), it is still very high. The IY for a second culture in smear negative episodes was 22.4%. Of the 733 culture positive SE, susceptibility results were available for 639 (87.2%). Of these, only 7 (1.1%) were resistant to isoniazid and rifampicin, precluding further analysis due to small numbers.

### HIV results

The HIV status and outcome were available only for treated patients that had consented to HIV testing: this represented 345 (13.6%) of the 2537 SE and 302 (41.2%) of the 733 culture confirmed SE. More than half of these 345 SE were HIV infected (192/345 = 55.7%). The yield of the first smear was 38.5% in HIV positive patients (74/192) compared to 51.0% (78/153) in HIV negative patients (chi-square = 5.34, p = 0.021). For HIV infected patients, the relative yield of 1 culture compared to 1 smear was higher than in HIV negative patients, with a relative yield of 2.1, an absolute yield of 43.2% and a NND of 2.3 (data not shown). However, these results should be interpreted with caution since the HIV status was known for only a small proportion of TB suspects.

## Discussion

When culture was added to the routine smear-based diagnosis of TB suspects in a high incidence area of South Africa, we found that 29.9% of 2537 SE were culture positive, compared to 20.0% for smear microscopy. The relative yield of culture-based diagnosis was almost double, consistent with the observation that microscopy typically has a sensitivity of about 50% when compared to culture [5-8]. In this high TB setting, one can obtain an additional TB diagnosis with just 8 cultures, or alternatively by submitting 19 second specimens for microscopy.

Although smear is less sensitive than culture, nevertheless it has excellent PPV in high incidence areas [3]. Our data indicate that while many patients can be detected by microscopy in a high-prevalence setting [22], the addition of culture-based diagnosis can detect many more. Moreover, the present study used the more sensitive fluorescent technique to define a microscopy-positive sample; the incremental yield of culture should be even greater in settings that do only light microscopy. Reviews on the sensitivity of smear compared to culture are based mainly on laboratory-based studies from developed countries, often with no description of the patient population studied [5,7,23]. If smears and cultures were performed and read exactly the same way on specimens from clinically similar patients in a low then in high TB prevalence area, we should observe similar sensitivity. However, because of the higher prevalence of positive smear and culture results observed in high endemic areas, the absolute and relative benefit will also increase, while the NND decreases. Paradoxically, countries with the lowest rates of TB typically do both microscopy and culture, while countries with the highest rates of TB often forego culture due to costs and lack of needed infrastructure. Our data supports recent statements by the WHO [9,24-27] and the International Standards for TB Care [28] on the use of culture in low income settings, recognizing that for each setting the incremental yield will need to be assessed against the incremental cost of culture-based testing.

In our study, almost 90% of positive cultures were positive on the first specimen, and the IY of the second culture was 10%. A lower yield (77.6%) was observed for the first specimen in smear negative SE compared to smear positive SE. These findings were also reported in studies that performed 3 cultures on patients [23,29-31]. However, finding 77% of culture positive patients with the first specimen is still significant and much more informative than a negative smear result. Table 5 compares our results to some other studies that have performed cultures in TB suspects and where results for first and second specimens were reported. The RY of 1C vs 1S was higher (2.7) in a study performed in HIV patients in Thailand and Vietnam [32], most likely because of the lower yield of light microscopy (Ziehl-Nielsen) compared to fluorescent microscopy.

**Table 5:** Yields and NND calculated on some other recent studies that have used culture based diagnosis in TB suspects

	**Our study**	**Our study SN SE**	**Monkongdee et al.**[32]	**Boehme et al.**[36]	**Ssengooba et al.**[35]
**Population**	**HIV pos and neg**	**SN in HIV pos and neg**	**All HIV pos**	**HIV pos and neg**	**All HIV pos and SN**
Pos on 1st S	377		36	561	
Pos on 2S	508		45		
Pos on 1st C	682	253	97	732	52
Pos on 2C	760	326	113		60
TB suspects tested	2537	2060	1060	1462	170
% Pos on 1st S	0.149		0.034	0.384	
% Pos on 2S	0.200		0.042		
% Pos on 1st C	0.269	0.123	0.092	0.501	0.306
% Pos on 2C	0.299	0.158	0.107		0.353
RY 2S vs 1S	1.3		1.3		
RY 1C vs 1S	1.8		2.7	1.3	
RY 1C vs 2S	1.3		2.2		
RY 2C vs 1C	1.1	1.3	1.2		1.2
AY 2S vs 1S	0.052		0.008		
AY 1C vs 1S	0.120		0.058	0.117	
AY 1C vs 2S	0.069		0.049		
AY 2C vs 1C	0.031	0.035	0.015		0.047
NND 2S vs 1S	19		118		
NND 1C vs 1S	8		17	9	
NND 1C vs 2S	15		20		
NND 2C vs 1C	33	28	66		21
NND of 1st S	7		29	3	
NND of 1st C	4	8	11	2	3

**Legend:** S = smear, C = culture, Pos = positive, Neg = negative, RY = Relative yield, AY = Absolute yield, SE= suspect episode, NND = number needed to diagnose. In the study by Monkongdee et al. [32], yields were reported using all culture positive cases as the denominator. The results shown here use all suspects tested as the denominator.

There were several limitations to our study. First, our analysis was done on 68.9% of the 3742 TB suspects with at least 1 smear and 1 culture result. This shows the challenges of obtaining 2 samples per TB suspect in regular programme settings. However, we were able to estimate the proportion of positives for the missing specimens using established formulas [19]. We excluded 15.4% of SE with contaminated culture results from our analysis in order to calculate the yield of first and second specimens accurately . Lower or similar contamination rates have been reported with the MGIT® system: 8.6% [33], 13% [32], 16.9% [34], 16.4% [35]. Despite excluding these contaminated results, we were nevertheless able to analyse a large number of SE. However, in real life, these results have an impact on costs and patient management [34]. Second, the HIV status was available only for treated patients that had consented to HIV testing, or 13.6% of 2537 SE. HIV voluntary counseling and testing (VCT) was offered at the clinic but low rates could be explained by the absence of routinely available antiretroviral therapy at the time of the study. Third, we were not able to compare the yield of the solid vs liquid culture media, as the individual results for these were not available. However, other studies have shown the higher yield of liquid vs solid culture [32-34]. Finally, some inconsistency rates in smear results were observed. When using all smear positive SE as a denominator, the proportion of SE positive on the first smear was 74.3%, lower than the 85% reported in a meta-analysis [8]. This may be due to the setting of the study in a regular programme. However, the incremental yield of culture over microscopy in this study is not due to false-positive cultures due to laboratory cross-contamination. We have assessed this possibility in another manuscript, using dummy samples, and found that the specificity of culture in this setting is 98.9%, which is comparable to other rates described in the literature [13].

Recently, a novel and simple nucleic-acid amplification test (Xpert® MTB/RIF) has been developed [36] and endorsed by WHO for global implementation [37]. Performing 1 Ziehl–Neelsen smear, 1 Xpert® MTB/RIF and 1 liquid culture on TB suspects yielded 38.4%, 46.2% and 50.1% positive results respectively. Compared to doing 1 smear, the RY, AY and NND would be 1.2, 7.8% and 13 for the Xpert® MTB/RIF and 1.3, 11.7% and 9 for culture [36]. However, as stated by WHO, the Xpert MTB/RIF technology does not eliminate the need for conventional microscopy culture and drug susceptibility testing [37]. The rapidity and simplicity of the method are clear advantages. However, sensitivity in smear negative patients remains an issue, with some reported sensitivities of 43.4% [38], 54.5% [39] and 72.5% [36]. Furthermore, drug susceptibility testing is still required to detect resistance to drugs other than rifampicin [37]. As the Xpert technique is implemented, initial studies show it can potentially be cost-effective compared to sputum smear diagnosis and clinical diagnosis in smear-negative cases [40]. Future studies could evaluate the cost-effectiveness of smear versus Xpert, as compared with smear versus culture, or even the integration of two of these tests. Depending on the prevalence of positive samples by new diagnostic techniques, the NND can be determined for either test, in order to predict the incremental gain in case detection, and ultimately, the impact on patient care.

## Conclusions

In conclusion, in a high TB burden setting, the use of culture methods can almost double the number of microbiologically-confirmed cases of TB, with just under 8 cultures required to detect 1 additional case.

## Appendix

Yield calculations (adapted from Rieder et al. [19]).There are 6 possible patterns among those who had a diagnostic examination (Ad) in a setting where 3 specimens are required: Px, NPx, NNP, NN, NN9 and N99, where P is a positive smear, x a subsequent result of no interest, N a negative result, and 9 a missing result. For our study, since we aimed to collect 2 specimens and since less than 10% of SE had ≥ 3 smears or 3 cultures done, we kept 4 patterns (Px, NP, NN and N9) and adapted the formulas accordingly.To calculate the incremental yields, the assumption was made that those with an N9 result had the same probability of being positive on the second smear as those with an NP result. Those (positive) missed by failing to do a second smear examination will be denoted as Md (Table 6).

**Table 6:** Definitions and formulas used for yield calculations

	**Description**	**Formula**
Ad	Number of SE who had specimens examined	Px + NP + NN + N9
	Observed # of P SE after 1 specimen	Px
Od	Observed # of P SE after 2 specimens	Px + NP
Sd1	Fraction of SE found positive on the 1^st^ specimen	Px/(Px + NP + NN + N9)
Sd2	Fraction of SE found to be negative on the 1st specimen but positive on the 2nd	NP/(NP + NN + N9)
Md	# of P SE missed by failing to do a 2nd specimen	Sd2 * N9
	Expected # of P SE after 1 specimen: same as Observed # of P SE after 1 specimen	
Ed	Expected # of P SE after 2 specimens (if all suspects had 2 specimens done)	Od + Md
	Expected proportion of P SE after 1 specimen: same as Sd1	
Rd	Expected proportion of P SE after 2 specimens	Ed/Ad
Fd1	Fraction for the potential IY from the 1^st^ specimen	Px / Ed
Fd2	Fraction for the potential IY from the 2^nd^ specimen	(Md + NPx) / Ed
OFP1	Overall fraction of P on 1^st^ specimen	Rd * Fd1
OFP2	Overall fraction of P on 2^nd^ specimen	Rd * Fd2
NND1	NND on 1st specimen: number of specimens needed to be examined to find one additional case	1/OFP1
NND2	NND on 2nd specimen	1/OFP2

**Appendix Legend.** # = Number, P = positive, SE = Suspect Episodes, NND = number needed to diagnose.

## Competing interests

All authors: no competing interests.

## Authors’ contributions

AMD and DC participated in all aspects from study design, data collection, analysis and writing. AB and MAB contributed to statistical analysis and writing. RW, SV, PvH, MAB contributed to study design, data analysis and writing. All authors read and approved the final manuscript.

## Pre-publication history

The pre-publication history for this paper can be accessed here:

http://www.biomedcentral.com/1471-2334/12/218/prepub
